# Mesenchymal Stromal/Stem Cells: A New Era in the Cell-Based Targeted Gene Therapy of Cancer

**DOI:** 10.3389/fimmu.2017.01770

**Published:** 2017-12-18

**Authors:** Faroogh Marofi, Ghasem Vahedi, Alireza Biglari, Abdolreza Esmaeilzadeh, Seyyed Shamsadin Athari

**Affiliations:** ^1^Department of Hematology, Tabriz University of Medical Sciences, Tabriz, Iran; ^2^Research Center for Food Hygiene and Safety, Shahid Sadoughi University of Medical Sciences, Yazd, Iran; ^3^Department of Genetics and Molecular Medicine, Zanjan University of Medical Sciences, Zanjan, Iran; ^4^Department of Immunology, Zanjan University of Medical Sciences, Zanjan, Iran; ^5^Cancer Gene Therapy Research Center, Zanjan University of Medical Sciences, Zanjan, Iran

**Keywords:** mesenchymal stem cells, gene therapy, cancer, vector, cell therapy

## Abstract

In recent years, in light of the promising potentials of mesenchymal stromal/stem cells (MSCs) for carrying therapeutic anticancer genes, a complete revisitation on old chemotherapy-based paradigms has been established. This review attempted to bring forward and introduce the novel therapeutic opportunities of using genetically engineered MSCs. The simplicities and advantages of MSCs for medical applications make them a unique and promising option in the case of cancer therapy. Some of the superiorities of using MSCs as therapeutic gene micro-carriers are the easy cell-extraction procedures and their abundant proliferation capacity *in vitro* without losing their main biological properties. Targeted therapy by using MSCs as the delivery vehicles of therapeutic genes is a new approach in the treatment of various types of cancers. Some of the distinct properties of MSCs, such as tumor-tropism, non-immunogenicity, stimulatory effect on the anti-inflammatory molecules, inhibitory effect on inflammatory responses, non-toxicity against the normal tissues, and easy processes for the clinical use, have formed the basis of attention to MSCs. They can be easily used for the treatment of damaged or injured tissues, regenerative medicine, and immune disorders. This review focused on the drugability of MSCs and their potential for the delivery of candidate anticancer genes. It also briefly reviewed the vectors and methods used for MSC-mediated gene therapy of malignancies. Also, the challenges, limitations, and considerations in using MSCs for gene therapy of cancer and the new methods developed for resolution of these problems are reviewed.

## Introduction

**Figure 1:**
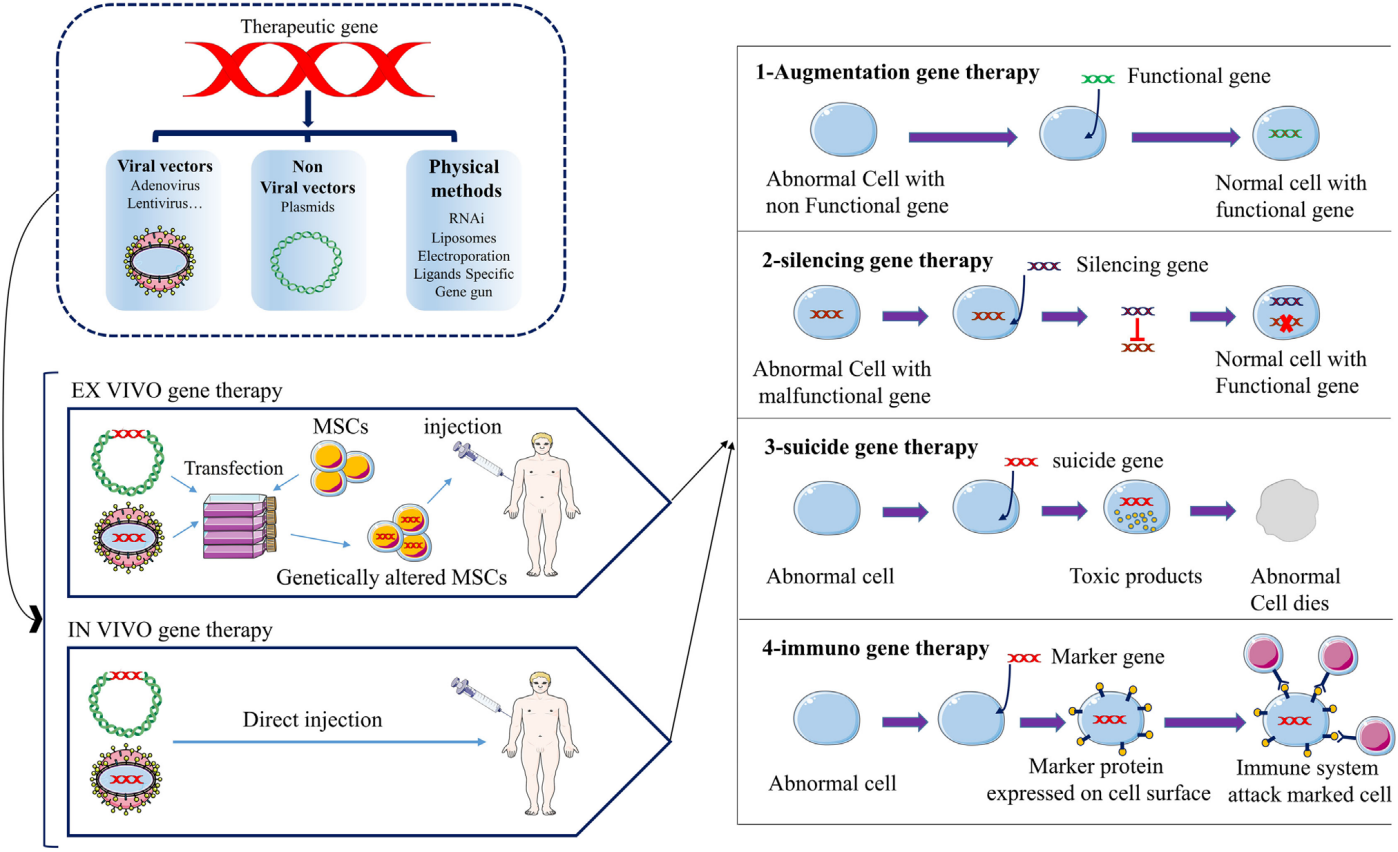
The schematic picture represents the strategies of anticancer gene therapy using mesenchymal stromal/stem cells (MSCs) as gene vehicles. Anticancer gene can be transferred into MSCs by three main groups of techniques; viral vectors, non-viral vectors, and physical methods. There are two approaches for gene therapy of cancer patients; *ex vivo, in vivo*. Four groups of anticancer gene therapies have been developed to date; augmentation-, silencing-, suicide-, and immune-gene therapy.

Generally, the establishment of cancer consists of three main stages, including development, growth, and metastasis. The most important actors in the initiation of cancer are the epigenetic changes and genetic mutations in proto-oncogenes, tumor suppressor genes, pro-apoptotic, anti-apoptotic, and cell cycle controlling genes. The tumor microenvironment provides tumor growth, chemotherapy resistance, immune escape, and tumor metastasis (1). Angiogenesis in tumor site is necessary for tumor growth and metastasis. Vascular endothelial growth factor (VEGF) and their receptors are one of the important molecules involved in angiogenesis. Today, many drugs act as the anti-angiogenesis treatments, most of which are monoclonal antibodies or tyrosine kinase inhibitors (1). However, tumor cells have different ways to survive from chemotherapies, including upregulation of alternative/compensatory pathways, resistance to chemotherapies and vasculogenic mimicry (1). Nowadays, gene therapy is the most promising novel approach for cancer therapy. Various approaches for gene therapy of cancer have been developed, including (1) engineered chimeric antigen receptor (CAR) T cells; CARs are engineered receptors with high specificity in identifying tumor-related antigens. They are connected to the inner domain of TCRs and have a great potential for specific activation of T cells against tumor cells. CAR-based therapies showed great anticancer efficacy in clinical evaluations especially against hematologic malignancies. (2) Tumor vaccine (DNA vaccine), DNA vaccines can establish anticancer immunity through the induction of expression of a specific gene. (3) Replacing the normal hematopoietic stem cells; the normal cells are transfected with specific chemotherapy-resistance genes then transplanted to the patient by bone marrow transplantation. The patient then receives chemotherapy in which the transfected cells remain but other cells die. (4) Clustered regularly interspaced short palindromic repeats (CRISPR)–Cas9 technology; CRISPR–Cas9 is a highly precise technique for genome editing. CRISPR–Cas9 and other similar nuclease-based genome editing systems, such as transcription activator-like effector nucleases (TALENs) and zinc finger nucleases (ZFNs), are used in gene therapy of cancers. The technique can target the desired section of human genome with the least off targets and as such can be used for therapeutic purposes in cancer therapy by specific targeting of the regulatory sequences, SNPs or oncogenes. (5) Therapeutic genes/agents delivery; in these methods, viral and non-viral vectors, tumor-tropic cells, and other micro-carriers are used for the transfer or expression of therapeutic genes, agents, or even oncolytic viruses. The delivered anticancer factors can be tumor suppressor genes, apoptosis-inducing genes, suicide genes, regulatory agents [e.g., RNA interference (RNAi), miRNAs], oncolytic viruses and immunological factors (e.g., cytokines) (1). Therapeutic genes can be transferred as the naked DNA or by using viral/non-viral vectors (2) (Tables 1–3). But the main disadvantage of this classical method of gene delivery is its generally non-selective nature. The non-specific targeting by administering the non-viral/viral vectors and their systemic distribution throughout the circulation can result in undesired adverse effects. Since the discovery of ability of transgene mesenchymal stromal/stem cells (MSCs) to selectively migrate toward the injured and tumor site(s), the gene therapy of cancers experienced a substantial leap ahead (3, 4). But despite the remarkable progress in the field of cancer gene therapy, there are two main obstacles still remaining, including the difficulty in obtaining a suitable drug carrier or transgene vehicle that can selectively migrate toward tumor location and the difficulty in discovering an eligible vehicle that can resist within the biased and hostile microenvironment of the tumor (5, 6). Therefore, huge efforts have been made to look for a proper vehicle (Table 3). The results hold out tremendous promise for MSCs due to their inherent tropism to tumor sites and immunomodulatory properties. MSCs are non-hematopoietic stem cells originally found within bone marrow but also present in other tissues, including adipose tissue, dental pulp, muscles, and skin (3, 4). Dental-tissue is an easily accessible source of stem cells. Dental-tissue-derived stem cells (DTDSCs) consist of six types of stem cells which can be isolated from the different anatomical locations on the dental tissues. However, it was shown that most of these DTDSCs lack a key feature of the MSCs which is multi-potency (7). Human MSCs pose a unique molecular fingerprint by expressing CD105, CD44, CD90, and CD73 but not CD79a, CD45, CD34, CD19, CD14, CD11b, and HLA-DR. They can be differentiated into various types of cells, including adipose tissue, bone tissue, and cartilage tissue. Although MSCs constitute a small population of bone marrow cells, they also play an apparent role in hematopoiesis (3, 8). In other words, using MSCs opens novel opportunities for a wide spectrum of clinical applications, such as the cell therapy, regenerative medicine, cancer gene therapy and treatment of graft versus host disease (Tables 1 and 2) due to their remarkable capacity for proliferation and differentiation, immunoregulatory effects, tendency toward the injured tissues and ease of isolation and expansion *in vitro* (3). In addition, low expression of costimulatory molecules by MSCs makes them nearly unidentifiable by immune system and as a consequence non-immunogen, empowering them for the stealthy movement and migration through the circulation. The low immunogenicity of MSCs enables them to be easily utilized for cell therapy even without HLA matching (9, 10). In this respect, it was found that after an intravenous injection, MSCs moved toward the damaged tissues or tumor site(s) without being attacked by the immune system as foreign invaders (Figure 1). Consequently, the mentioned unique properties possessed by the engineered/modified MSCs can be utilized with high levels of success as the carriers of the genes encoding for anticancer molecules (4, 6, 11) (Tables 1 and 2). The strategies applied for the anticancer genes/agents delivery are based on the following principles (1) (Figure 1): (1) Augmentation gene therapy which includes: (a) expressing a gene to prompt apoptosis (e.g., TRAIL, mda-7, Caspases and selective short interfering RNA (siRNA)/microRNA (miRNA)-mediated blocking of anti-apoptotic genes), (b) improving tumor sensitivity to chemo/radiation therapy, (c) introducing a tumor suppressor gene (e.g., P53, Rb, p16INK/CDKN2, and PTEN). (2) Gene silencing therapy: inhibition of expression of an oncogene (C-MYC and K-Ras) by employing an antisense (RNA/DNA). (3) Suicide gene therapy: delivery of a converting enzyme to the site of tumor that convert non-toxic prodrug to the toxic drug. (4) Immuno-gene therapy: increasing the immunogenicity of the tumor cells/tissue to stimulate immune cell response against tumor (1) (Figure 1). The major hallmark explained for MSCs as the cell carriers is the ease of introducing new therapeutic genes into their genetic material and subsequently the simplicity of utilizing them for *in vivo* trials (3, 12). Recent studies have shown the successful application of lentivirus, retrovirus, or plasmid as the operational vectors to transfer genes into MSCs (13, 14) (Table 3). Moreover, MSCs are capable of being reprogrammed for transporting therapeutic molecules/proteins in the same manner that they can carry the therapeutic genes. This special attribute helps clinicians to overcome the adverse effects associated with the direct injection of drugs or other therapeutic molecules. This is of great importance when the biological properties and adverse effects of therapeutic molecules are considered, thus the positive role of engineered MSCs in preventing the redundant effects might be highly appreciated (4, 6). Furthermore, there have been an increasing number of encouraging evidences indicating the successful utilization of MSCs as the vehicles of therapeutic genes in neurodegenerative disorders, cancer, cardiovascular diseases, bone tissue fractures/defects, and various organs abnormalities (e.g., in the liver, pancreas, lungs, and kidneys) (4, 6, 12) (Tables 1 and 2).

**Table 1:** A list of cytokines, chemokines, prodrugs, and other agents with the anticancer properties that were transferred (or can be transferred) into the mesenchymal stromal/stem cells (MSCs) and integrated into genomic material then delivered by the cell toward the tumor site(s)/cells. The reports listed in three categories including *in vitro* studies, preclinical (mouse *in vivo*) studies, and clinical trials (human *in vivo*).

Factor	Application	MSCs	Host	Vector	Tumor model	Result	Reference
***In vitro* (cell lines)**							
IFN-γ	Immunostimulatory, apoptosis inducing	Human BM-MSC	*In vitro*	Adenoviral (Ad) vector	*In vitro* (leukemia)	Inhibits the proliferation of K562 cells and induces apoptosis	(15)
Oncolytic viruses	Destroy tumors by viral replication	Human BM-MSC	*In vitro*	Recombinant Ad vectors	Orthotopic (breast, lung, ovarian). Metastasis (breast)	Increased the survival of tumor-bearing mice. Further, decreased the tumor growth	(16–18)
IL-27	Reduction of inflammation and autoimmune diseases	Human AD-MSCs	In vitro	Lentiviral vector (LVs)	*Ex vivo* gene therapy	The LVs did not impact MSC characteristics and inhibited the inflammatory responses	(19, 20)
IL-18	Stimulates innate immunity and Th1–Th2-mediated responses, antitumor effect, reduces tumorigenesis, induces apoptosis, and inhibits tumor angiogenesis	Human UC-MSCs	*In vitro*	LVs	MCF-7 and HCC1937 cells	IL-18-producing MSCs significantly suppressed the proliferation, migration and invasion of the MCF-7, and HCC1937 breast cancer cells	(21)
IL-7	Essential role in the survival and homeostatic proliferation of peripheral naive T cells	Human BM-MSC	*In vitro*	Retroviral Vector	*In vitro* co-culture of MSCs with naive T cells	IL-7-MSCs have a dose-independent effect on naive T-cell survival while exerting a dose-dependent effect on activation/proliferation	(22)
IL-35	Reduction of inflammation and autoimmune diseases	Human WJ-MSCs	*In vitro*	LVs	Mouse splenocytes	Induces the proliferation of Treg cell, reduces the activity of Th17 and Th1	(23)
**Preclinical (animal model)**							
IFN-α	Immunostimulatory, apoptosis inducing, and anti-angiogenic	C57BL/6 mice BM-MSC	C57BL/6 mice	(rAAV)	Metastasis (melanoma)	Increased apoptosis and decreased proliferation and angiogenesis of tumor	(24)
IFN-β	Induces differentiation	Human BM-MSC	Female C.B-17 SCID mice	Ad vector	Metastasis (prostate, breast, melanoma)	A significant reduction in tumor volume following IFN-β expressing MSC therapy	(25–27)
	S-phase accumulation, apoptosis	Human BM-MSC	Male athymic nude mice (nu/nu)	Ad vector	Orthotopic (glioma)	Injected MSC-IFN-β Cells suppressed the growth of orthotopic metastases	(11)
IL-2	Immunomodulation	Rats BM-MSC	Male Fisher 344 rats	Ad vector	Orthotopic (glioma)	Augmented the antitumor effect and prolonged the survival of tumor-bearing rats	(28)
IL-12	Activates CTLs and NK cells and stimulates production of IFN-γ	Mouse BM-MSC	Female syngeneic C57BL/6 and BALB/c inbreed mice	Ad vector	Subcutaneous (melanoma, Hepatoma, Lung cancer)	Systemic administration of MSC/IL-12 reduced the growth of 786- 0 RCC and significantly prolonged survival of tumor-bearing mice	(29, 30)
CX3CL1	Activates CTLs and NK cells	BALB/c, C57BL/6 BM-MSC	Female C57BL/6 (H-2b), BALB/c (H-2d), and BALB/C nude mice	Ad vector	Metastasis (melanoma, colon)	Suppressed the growth of multiple tumor metastases and prolonged survival in mice	(31)
GCV/HSV-tk	Prodrug conversion	Human AT-MSC	Athymic nude mice (Balb/c-nu/nu)	Retroviral vector	Subcutaneous, orthotopic (glioma)	Profound bystander effect on neighboring tumor cells *in vitro* by formation of gap junctions between cells. Selectively targets and kills the tumor cells by TK-MSC/GCV cells based on GJIC machinery	(32, 33)
5-FC/CD	Prodrug conversion (5-FC to 5-FU)	Human AT-MSC	Athymic nude mice (Balb/c-nu/nu)	Retroviral vector	Subcutaneous (melanoma, Colon)	Complete tumor regression in a dose-dependent manner or did not even allow the establishment of the tumor	(34–36)
NK4	Inhibits angiogenesis and promotes apoptosis	Female BALB/c mice BM-MSC	BALB/c mice	Ad vector	Metastasis (colon)	Significantly prolonged survival of the C-26 tumor-bearing mice by inhibiting tumor-associated angiogenesis and lymphangiogenesis and inducing apoptosis of the tumor cells	(37)
TRAIL	Induces apoptosis	Human BM-MSC	Nude mice	LVss	Subcutaneous (breast) Metastasis (breast) Orthotopic (Glioma)	TRAIL-expressing MSCs were able to reduce tumor growth and metastases	(38–40)
TNF-α	Induction of apoptosis and necrosis	Human AT-MSC	Nude mice (Balb/c-nu/nu)	Recombinant retroviral vector	Melanoma (A375), breast carcinoma (SKBR3, MDA-MB-231), colon carcinoma (HT29), ovarian carcinoma (SKOV3), and glioblastoma (U87-MG) cells	Induces apoptosis of tumor cell lines *via* caspase 3/7 activation and inhibits the tumor cell proliferation *in vitro*. In mice model, the tumor mass inhibition was up to 97.5%.	(41)
CCL5	Recruits MSCs	C57BL/6 mice BM-MSC	C57BL/6 mice	Ad vector	Orthotopic Pancreatic Carcinoma	HSV-Tk transfected MSCs led to a significant reduction of primary pancreatic tumor growth and metastases	(42)
TSP-1	Anti-angiogenic effects	Human BM-MSC	SCID mice	LVs	Human glioblastoma (GBMs, LN229-mCherry-Fluc tumor)	Inhibits tumor progression and extends survival of mice bearing highly vascularized GBM	(43)
PE cytotoxin	Blocks protein synthesis, antitumor agent	Human BM-MSC	SCID mice	LVs	Glioblastoma	Long-term survival of mice	(44)
IL-24	anticancer agent, anti-angiogenic, induction of apoptosis and cell cycle arrest	Human UC-MSCs	BALB/c nu/nu mice	Ad vector	A549 lung cancer cells	IL24-MSCs promote apoptosis, inhibit proliferation, and decrease the vascularity of xenograft tumors	(45)
	Hypothesized suppressor effect on the tumor growth, anti-angiogenic effect, induction of apoptosis and cell cycle arrest	–	Athymic nude mice/SCID mice	Ad vector	Thyroid carcinoma/Hodgkin’s lymphoma	Theoretically IL24 can promote apoptosis *via* blocking the anti-apoptotic regulators and activation of TRAIL and JAK-STAT pathway, inhibit proliferation	(46, 47)
IL-25	Hypothesized pro-apoptotic action	Mouse BM-MSC	C57BL/6 mice BM-MSC	Lipofection	Pancreatic cancer	Hypothesized induction of apoptosis in cancer cells	(48)
Soluble IL-1 receptor–like–1 (sST2)	Decoy receptor for IL-33 in order to enhance immunoregulatory and anti-inflammatory effects of MSCs	Human AT-MSC	male BALB/c mice	LVs	Endotoxin-Induced Acute Lung Injury; male BALB/c mice challenged with intranasal instillation of LPS	Remarkable reduction in lung airspace inflammation. Mitigation of apoptosis, and minimal inflammatory cell infiltration. Hampered IL-33, Toll-like receptor (TOL)-4, IL-1b, and IFN-g induction, but up-regulated IL-10 expression in the injured lungs	(49)
IL-10	Immune suppressor. Prevent transplant rejection.	Human BM-MSC	Inbred male DA (RT1n) and Lewis (LEW) (RT1l) rat	lentivirus-based plasmid construct	liver allograft in mice model	Long-term survival of recipient’s mice. IL-10- MSCs therapy can overcome rejection of liver transplant	(50)
**Clinical trial (human)**							
HSV-Tk, Ganciclovir	Suicide gene therapy	Autologous BM-MSCs	Human (Clinical trial)	Gamma-retroviral	Advanced, recurrent or metastatic gastrointestinal or hepatopancreatobiliary adenocarcinoma	Phase I/II clinical trial done and Phases III/IV are underway	(51)
HGF	Protects against fibrosis. Pro-angiogenic activity	Autologous BM-MSCs	Human (Clinical trial)	Plasmid HGF	Four patients with pulmonary silicosis	Chest distress gradually ameliorated at six months post-therapy, accompanied by the significant improvement of pulmonary function	(52)

*rAAV, recombinant adeno-associated virus; WJ, Wharton’s Jelly*.

**Table 2:** Gene-directed enzyme pro-drug therapy (GDEPT) of cancers using the various types of mesenchymal stromal/stem cell (MSCs).

Factor	Host	MSCs	Vector	Tumor model	Reference
TK (GCV)	Nude mice	BM-MSCs of rat	Non-viral	Pulmonary melanoma metastasis	(53)
	Nude mice	Human BM-MSCs	Retrovirus	Human glioma and rat glioma	(54)
	Nude mice	Human BM-MSCs	Adenovirus	Human glioma	(55)
	Nude mice	BM-MSCs of rat	Adenovirus	Lung metastases	(56)
	Nude mice	Human BM-MSCs	Baculovirs	Human glioma	(57)
	Nude mice	Human ASCs	Retrovirus	Glioblastoma multiforme	(33)
	Mice Murine	Mice BM-MSCs	Non-viral	Pancreatic carcinoma cells of murine	(42)
	Nude mice	Murine BM-MSCs	Non-viral	Hepatocellular carcinoma cells of human	(58)
	Nude mice	Murine BM-MSCs	Non-viral	Orthotopic pancreatic breast cancer of mouse	(59)

CDb (5-FC)	Athymic nude mice	Human BM-MSCs	Non-viral	Gastric cancer cells of human	(60)
	Nude mice	Rat BM-MSCs	Adenovirus	Rat glioma cells	(61)
	Male Fisher 344	Rat BM-MSCs	Adenovirus	Rat glioma cells	(61)

CDy::UPRT (5-FC)	Male SpragueeDawley	Human ASCs	Retrovirus	Rat glioblastoma cells	(62)
	Athymic nude mice	Human ASCs	Retrovirus	Human prostate cells	(36)
	Athymic nude mice	Human ASCs	Retrovirus	Human melanoma cells	(34)

**Additional prodrug enzyme combinations**					

CE (CPT-11)	Rat Fischer 344 female	Human ASC	Plasmid	Rat glioma cells	(63)
CYP2B6 (CPA)	Female nude mice	Murine	Retrovirus	Human glioma cells	(64)

*BM-MSCs, bone marrow-derived mesenchymal stem cells; AT-MSCs, adipose tissue mesenchymal stem cells; i.v., intravenous; s.c., subcutaneous; dTRAIL, dodecameric human TRAIL; hASCs, human adipose-derived stroma and stem cells; NSCs, neural stem cells, CDy::UPRT, fusion yeast cytosine deaminase::uracil phosphoribosyltransferase gene*.

**Table 3:** The features, advantages, and disadvantages of the most used viral and non-viral vectors for gene transferring into the target cells (65–78).

Vector type	Characteristics	Advantages	Limitation	Tropism	Host genome	Transgene expression	Packaging capacity
Adenovirus	36 kb dsDNA Non-enveloped Non-integrating	Large genome Easy to produce high titer Infects many cell types	High immunogenicity	–	–	–	–
Retrovirus (lentivirus)	8 kb ssRNA Enveloped Integrating	Large genome High infection efficiency Stable gene transfer	Insertional mutagenesis	Dividing and non-dividing cells	Integration in genome	Stable	8 kb
Adeno-associated virus (AAV)	4.7 kb ssDNA Non-enveloped Non-integrating	Low immunogenicity Infects many cell types Long-term gene transfer	Small genome Low transduction efficiencies	Dividing and non-dividing cells	No integration	Stable in non-dividing cells	5 kb
Herpes virus saimirii (HVS)	-	Transduction efficiencies of up to 95%	Safe replication-deficient HVS vector	–	–	–	–
Oncogenic retroviruses	Moloney murine leukemia virus (MoMLV)	Large genome	Shorter expression time Insertional mutagenesis	–	–	–	–
Baculovirus	Viruses are derived from an insect: *Autographa californica* multiple nucleopolyhedroviruses	Replication-defective Large genome High infection efficiency	Shorter expression time	–	–	–	–
Plasmid	To clone a DNA insert with maximum size of 15 kb	–	–	–	–	–	–
Non-viral	Calcium phosphate, liposomes, niosomes Nanoparticles, Spermine–pullulan	Ease of synthesis, cell/tissue targeting, low immune response, and unrestricted plasmid size	Shorter expression time lower transfection efficiency	–	–	–	–

## Part I: MSCs in the Field of Cancer Therapy

### The Mechanisms of MSCs Homing to Tumor Tissue

**Figure 2:**
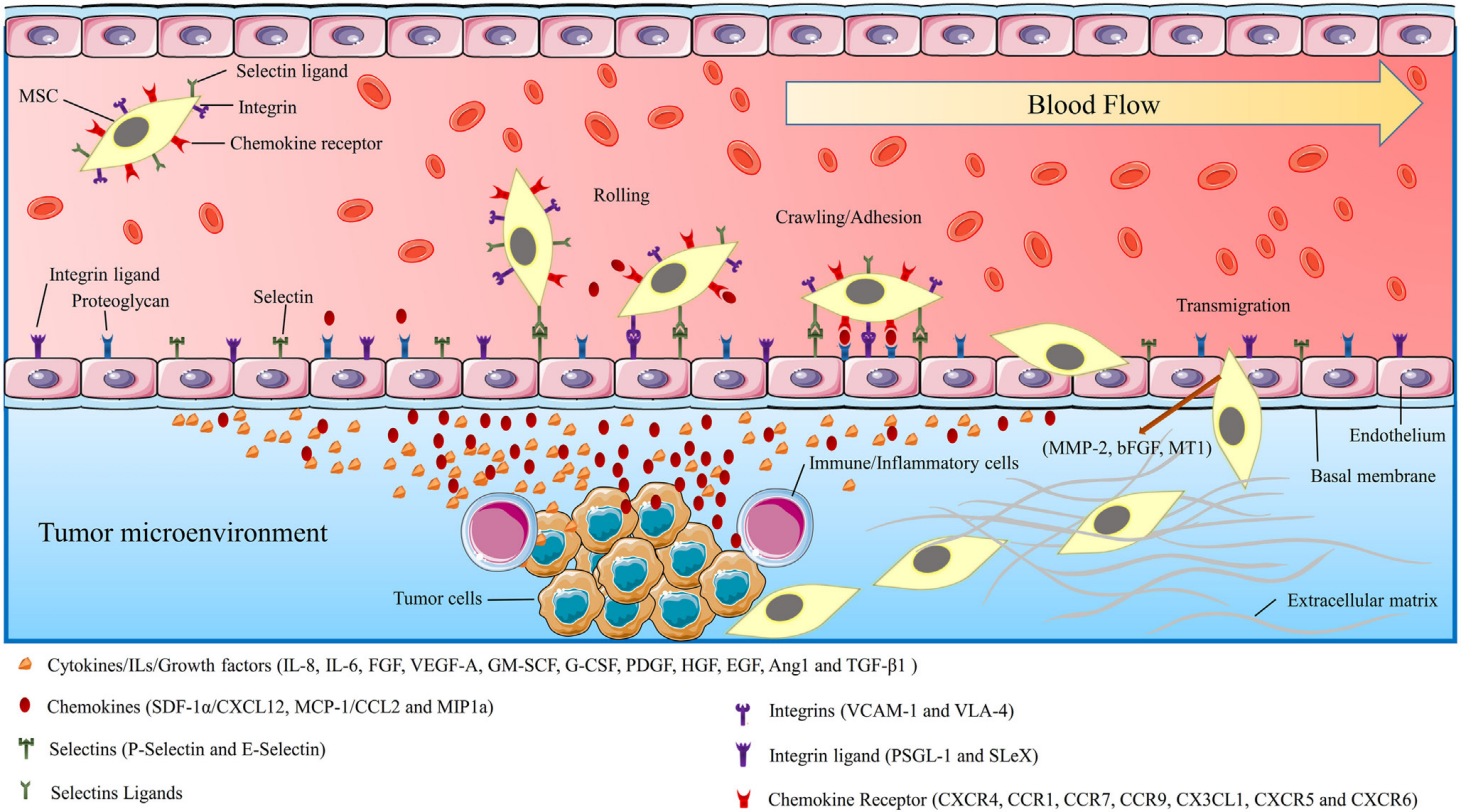
The schematic picture shows the mechanism of mesenchymal stromal/stem cells (MSCs) migration/homing toward injured/cancer tissues. MSCs moving toward target tissue can be characterized by three stages: flowing through blood-flow, moving toward chemoattractants, and adhesion by surface receptors, translocation from blood flow toward injured/cancer tissue. The figure also demonstrates the endothelial–MSCs interactions, receptors, and the chemokines which are involved in MSCs migration procedures. The tumor-affected endothelial cells produce selectins, integrin ligands, and chemokines. These secretory stimulants and chemoattractants recruited MCSs to the tumor site.

Many studies predicated that the mechanism by which MSCs migrate to the tumor site(s) is associated with the biologic characteristics of the tumor microenvironment (69, 70). Tumor cells resemble a chronic inflammation within the tumor microenvironment by generating high concentrations of inflammatory chemokines and growth factors (4, 6, 71). It is suggested that the selective migration of MSCs to the tumor site is linked to the high local concentrations of dozens of chemoattractants and growth factors that are secreted by tumor cells and inflammatory cells (6, 69, 72). Some of the most well-known chemokines associated with tumor progression and angiogenesis are fibroblast growth factor, stromal-derived growth factor-1α (SDF-1α/CXCL12), vascular endothelial growth factor-A (VEGF-A), granulocyte–macrophage colony-stimulating factor (GM-SCF), granulocyte colony-stimulating factor (G-CSF), platelet-derived growth factor (PDGF), epidermal growth factor (EGF), angiopoietin-1, monocyte chemoattractant protein-1 (MCP-1/CCL2), hematopoietic growth factor, transforming growth factor beta-1 (TGF-β1) IL-8, IL-6, and urokinase-type plasminogen activator (6, 73, 74). Recently, it was revealed that CXC chemokine receptor 4 (CXCR4) is one of the most important chemokine receptors responsible for the recruitment and tumor tropism of MSCs (75). Other chemokines and their receptors with the fundamental role in tumor tropism of MSCs are CCR1, CCR7, CCR9, CX3CL1, CXCR5, and CXCR6 (6, 10, 76) (Figure 2). Recent studies have shown that the mechanism of MSCs homing to the tumor site is very similar to that elicited by WBCs, directing them toward tumor and inflammation site(s). A variety of molecules such as integrins, selectins, and chemokine receptors are involved in the migratory process by leukocytes to the tumor site and damaged tissue. Interestingly, all of these molecules are also highly expressed in MSCs. In addition, a wide range of growth factors, chemokines, adhesion molecules, and toll-like receptors which are expressed by MSCs are increasingly thought to be responsible for tumor tropism of MSCs (6, 69, 72, 76) (Figure 2).

### Increasing the Efficiency of Tumor Tropism of MSCs

Theoretically increasing the tumor tropism efficiency of MSCs to its maximum possible level should cause the MSCs to reach the capability of effectively finding their way to not only the primary tumor location but also to the metastases locations (72). The homing property of MSCs varies in different types of tumors, therefore, researchers have focused much of their attention on establishing the precise and comprehensive modalities to maximize MSCs homing properties toward various types of solid tumors (4, 6, 77). Thus, it seems that working on finding innovative methods to stimulate the upregulation of expression of the surface molecules with a role in adhesion of MSCs to the tumor endothelium should be the best option to develop a tumor-tropism boosting method effective for therapeutic anticancer gene delivery (4, 6). The engineered MSCs, expressing the epidermal growth factor receptor, have shown considerably higher tumor tropism (6, 78). Furthermore, on the other side, alterations in the tumor niche are subjected in numerous examinations with the final goal of enhancing the MSCs recruitment to the tumor site(s) (4, 6). Previous studies have shown that tumor microenvironment is fulfilled with huge amounts of diverse kinds of chemotaxis-inducing and inflammatory molecules resembling a chronic inflammatory response that can efficiently increase the tumor tropism of MSCs (4, 6, 74). A series of methods have been evaluated for their potential to stimulate the tumor cells to release chemoattractants. For instance, low-dose irradiation of tumor can increase the recruitment of MSCs to the tumor site(s) (79). Researchers have found that irradiation increases the apoptosis and stimulates the danger signals. The danger signals thereby induce the production of inflammatory cytokines such as PDGF, TNFα, CCR8, and CCR2 within the tumor microenvironment, and consequently enhance MSCs swarming into the tumor location(s) (79).

### Reprogramming MSCs for Targeted Cancer Therapy

**Figure 3:**
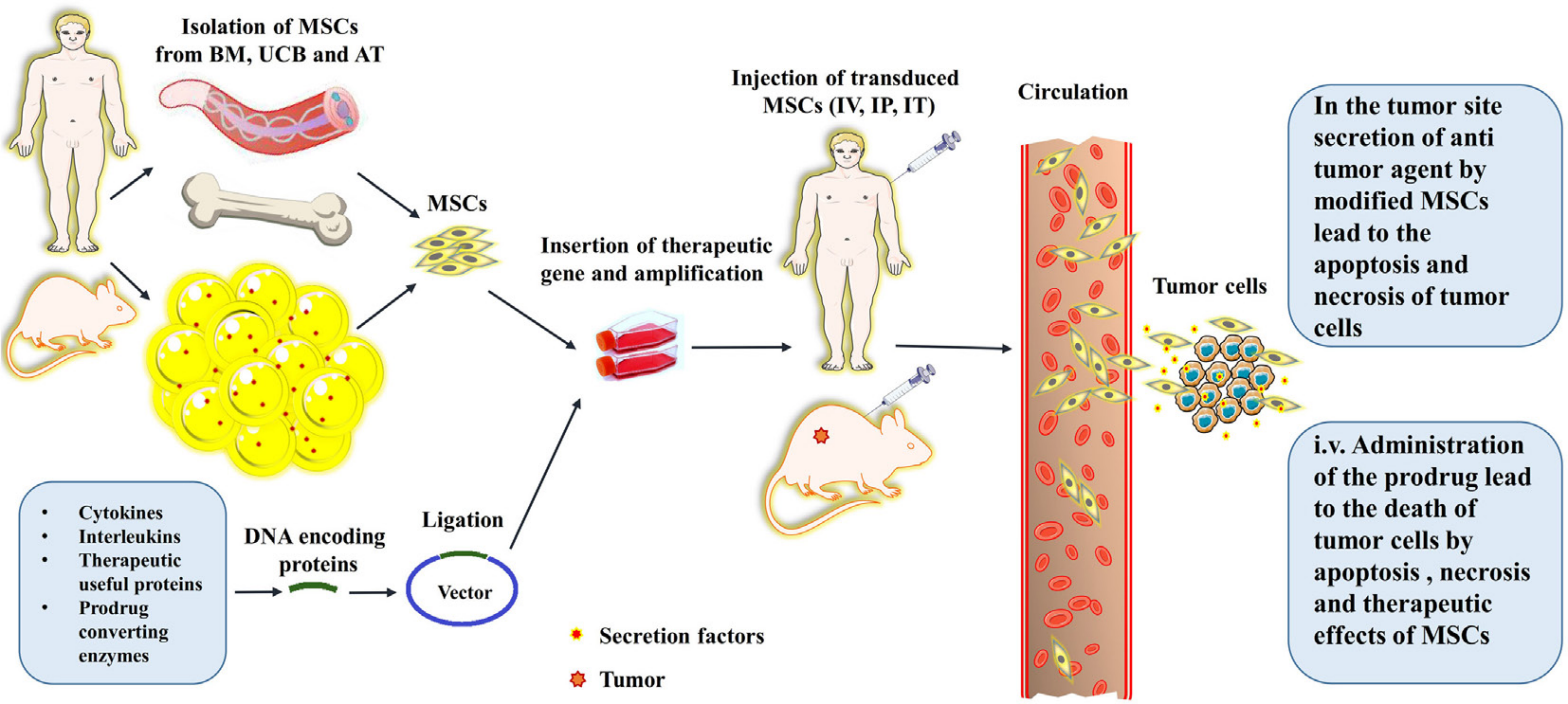
A schematic picture depicts the procedures for the isolation, culture, gene transfer, and *in vivo* administration of the mesenchymal stromal/stem cells (MSCs). The schema also shows the routes that the MSCs traverse toward their target; tumor sites, and cancerous cells. The abbreviations on the figure include the following: BM, bone marrow; UCB, umbilical cord blood; AT, adipose tissue; IV, intravenous; IT, Intrathecal; IP, Intraperitoneal.

Mesenchymal stromal/stem cells were utilized for the first time in 2002 for targeted-delivery of INF-β gene in the treatment of cancer. The transgene MSCs carrying INF-β gene were injected to the tumor-bearing mice which resulted in a significant decrease in tumor growth and accordingly a considerable increase in survival rate of mice in comparison to the control group (25) (Table 1). These encouraging results have paved way for the application of engineered MSCs for targeted delivery of genes and therapeutic drugs for treatment of cancers. Interleukins as the key regulators of inflammation and immune system functions are suitable options for therapeutic use in MSC-based gene therapy of cancers (Table 1). Engineering MSCs to secrete IL-12 in tumor-bearing mice resulted in very promising outcomes. IL-12 activates the cytotoxic lymphocytes and NK cells, induces the apoptosis, and prohibits the metastasis of tumor cells (29) (Table 1). In addition, other therapeutic genes encoding for regulatory proteins and immunomodulatory cytokines such as CX3CL1, INF-β, INF-α, INF-γ, IL-2, hepatocyte growth factor antagonist NK4, pigment epithelium-derived factor, TRAIL, and TNF-α have shown antitumor effect similar to that by IL-12 (3, 4, 6) (Table 1). TRAIL is the ligand for death receptors which are overexpressed on the surface of tumor cells. TRAIL can initiate the caspase-mediated apoptosis leading to inhibition of tumor growth (4). Surprisingly, MSCs are nearly resistant to TRAIL-induced apoptosis due to very low expression of death receptors (4). Generally, other normal cells are also resistant to TRAIL-induced apoptosis due to the absence or low expression of TRAIL receptors on their surface. Therefore, TRAIL-directed death induction can be of great advantages to the designing of a selective cancer therapy. In this respect, TRAIL-secreting MSCs have been used in diverse models of cancers with outstanding antitumor effects (4). TRAIL-expressing extracellular vesicles (EVs) derived from MSCs are also used as the selective therapy against 11 cell lines with promising antitumor effects and without considerable cytotoxicity against normal human bronchial epithelial cells. These MCS-derived EVs can home toward tumor site(s) then target the cancer cells by a target-specific action (80). A novel engineered oncolytic adenovirus with improved affinity to the host cells has also been used to treat pancreatic ductal adenocarcinoma (PDA); a malignant and aggressive cancer with poor prognosis. The oncolytic virus which was also carrying a TRAIL gene in its genetic code could efficiently infect and lyse the tumor cells while simultaneously inducing the apoptosis of non-infected tumor cells. In this method, an engineered Ad vector containing the TRAIL coding gene was transferred into the MSCs (Ad-TRAIL-MSCs) then the Ad-TRAIL-MSCs were administered to mouse models of PDA. The tumor growth was strongly hampered in mice receiving Ad-TRAIL-MSCs. The method did not show any toxicity or side effects and the anticancer action was tumor-specific due to MSCs selective homing into the tumor site(s) (81). Recently, suicide-gene therapy has been considered as an effective method in the treatment of many malignant and metastatic cancers (4). In order to further examine these suicide-inducing techniques, a cytokine-mediated death-boosting method was hypothesized based on using the IL-25 (aka IL-17E). It was assumed that if the gene encoding for IL-25 be transferred into the MSCs then these cells will release the IL-25 within the tumor environment. Migration of these MSCs to the tumor site(s) can exert an effective sequestration of the tumor growth *via* induction of cell death (48) (Figure 1; Table 1). However, MSCs themselves demonstrate strong and direct anti-inflammatory effect (3). In this respect, the suppressing effect of MSCs on the unbalanced and overactive immune system in a disease called Behçet was also hypothesized. In Behçet’s disease, mucosal ulcers formed due to the hyper-reactive response of the immune system by excess production of pro-inflammatory cytokines (82). In another method called gene-directed enzyme prodrug therapy (GDEPT), at the first stage, the genes encoding the pro-drug activating enzymes are transferred to the tumor site using MSCs as the cell vehicles (Table 2). Subsequently, inactive and non-toxic prodrug is injected to the body. Then, pro-drug is catalyzed by the enzymatic cleavage to the activated form within the tumor environment. At the last stage, cytotoxic metabolites derived from injected and catalyzed pro-drug are released to the tumor microenvironment causing apoptosis, necrosis and death of the tumor cells (83) (Figure 3). Following the lysis and death of tumor cells, huge amounts of lysed cell remnants and toxic substances are leaked from dying, necrotic or apoptotic cells within the tumor microenvironment. The toxic molecules initiate a cascade of anticancer effects by immune system. The immune system reacts to these danger signals by recruiting the effector cells such as cytotoxic T cells and macrophages toward the tumor. Afterward, these effector cells produce various cytokines causing even more recruitment of immune cells to the tumor site. Recruitment of cytotoxic and effector cells lead to more efficient death and apoptosis of cancer cells and reduction of tumor volume (83). The most common enzyme-prodrug complexes that are used in animal models of various tumors are comprised of herpes simplex virus thymidine kinase complexed with Ganciclovir (HSV-TK/GCV system), CD with 5-fluorocytosine (5-FC), CE with irinotecan (CPT-11), and cytochrome P450 with cyclophosphamide or ifosfamide (83) (Table 2). Furthermore, engineered MSCs are also used in virotherapy of cancers with the help of the oncolytic viruses. MSCs can transport the oncolytic viruses to the tumor location. The efficacy of genetically engineered MSCs carrying oncolytic viruses has been confirmed in experiments. These virus carrying-MSCs can cause a substantial mitigation in tumor growth and metastasis, and therefore a good enhancement in the survival ratio in tumor-bearing animals (4).

### MSCs Interact with Tumor Cells, Acting Like a Double-Edged Sword

Mesenchymal stromal/stem cell-based delivery approaches of anticancer genes/agents have recently gained much attention from the scientific community as an innovative and exciting strategy for cancer treatment. However, there are also some drawbacks which the investigators encountered during the MSC-researches; it was found that stromal cells, pericytes, and endothelial cells exert a supportive role in tumor growth and progression by producing several growth factors such as TGF-β, PDGF, EGF, VEGF-A, and IL-8 (5, 6). In addition, it was suggested that following the accumulation of MSCs in tumor site, they can differentiate to cancer-associated fibroblasts (CAFs) or pericytes. Then, the CAFs can provide a structural support for the tumor microenvironment which lead to the sustained growth of the tumor (6). It was found that MSCs can support the invasion, growth, and metastasis of breast cancer cells by producing CCL5 and interestingly in return, the MSCs themselves are supported by the cancer cells (5, 6, 69). Despite these pitfalls, MSCs exert strong anticancer effects through inhibition of phosphorylation of AKT, leading to induction of apoptosis and prevention of cell cycle progression (6). In addition, MSCs can inhibit tumor cell growth by producing DKK-1, an important WNT antagonist (6). Moreover, MSCs showed inhibitory effects on various types of gastrointestinal cancers such as esophageal cancer, gastric cancer, and pancreatic carcinoma (10). The major anticancer mechanism by which is responsible for therapeutic effect of MSCs in gastrointestinal cancers is down-regulation of WNT signaling (10, 84). However, contradictory results have been reported about MSCs effect on different types of gastrointestinal cancers (10). Collectively, it was cleared that there is a bidirectional and mutual interaction between MSCs and tumor cells which sometimes may lead to a huge uncertainty in the applicability of MSCs in therapy of cancers. Therefore, it is necessary to engineer MSCs for proper expression of antitumor genes and upgradation of tumor tropism capacity to prevent unexpected results.

## Part II: Gene Delivery Methods

### The Methods for Gene Delivery into MSCs

The most important feature of MSCs as the cellular vehicles for gene delivery is the high capacity to be genetically manipulated *in vitro*. The genetic manipulation can be done using various vectors including lentiviral, retroviral and plasmid vectors (Table 3). Nevertheless, the Ad vectors have constituted the majority of viral vectors that are used for transduction of MSCs (3, 4). However, the therapeutic anti-cancer genes can be transferred into the MSCs directly without using viral or non-viral vectors (3) (Figure 1). The resultant transgene MSCs can also produce the therapeutic agent similar to vector-modified MSCs without some unwanted disadvantages such as the malignant transformations due to wrong genetic modifications (3).

### Viral Vectors

Viral vectors such as retroviral, lentiviral, Ad, and adeno-associated virus vectors have been extensively used for MSCs transduction (65) (Table 3). Additionally, viral vectors have shown a high potential for direct gene transfer into the tumor location with no requirement of being transported into a transgene cell which leads to a high ratio of transduction of target/tumor cells (2). However, we are faced with the possible severe adverse effects due to the systemic distribution of viral vectors. In addition, to improve the efficiency of gene transduction, a modified vector called fiber-mutant adenovirus vector has been developed. The vector could successfully moderate the tumor growth by transferring a gene encoding for an antitumor agent to the tumor cells (66). Also, PEG-modified viral vectors are refractory to the neutralizing antibodies and capable of accumulation at the tumor site(s) (66). Nevertheless, as mentioned above, although numerous studies used different viral vectors to target the tumor cells but application of this system has ceased in some of the clinical trials because some unexpected side effects, such as oncogenicity, immunogenicity, and toxicities, were observed following the administration of the non-improved vectors (66).

### Ad Vectors

Adenovirus vectors are the most commonly used vectors for transduction of MSCs (Tables 1 and 2). The ratio of successful transduction is closely associated with the expression of the matching receptors on target cells such as coxsackievirus and adenovirus receptors (CARs) (66). Accordingly, gene delivery using intact adenovirus vectors encounters a very low efficiency because the matching CARs are expressed at low levels on the surface of MSCs as well as in most types of the tumors (Table 3). Therefore, extensive efforts have been done to improve the efficiency of transgene delivery of Ad vectors by enhancing the infectivity of Ad vectors *via* the modifications done on the viral capsid and fibers (66, 85). In this regard, chimeric Ad vectors were designed with high efficiency for transgene delivery into stem cells (Table 3). Recently, a capsid-modified adenovirus with the ability to bind desmoglein-2, a widely expressed cell surface marker, was developed. The resulting Ad particles could infect both cancer cells and MSCs with high efficiency (85). In addition, application of a fiber-modified adenovirus vector comprising a RGD motif—in the HI loop of the fiber knob domain targeting the surface adhesive integrins on the MSCs increased the transduction efficiency (65, 66).

### Lentiviral Vectors (LVs)

Lentivirus vectors are the second most widely used vectors in MSC-based cancer gene therapy (Tables 1 and 2). These vectors have some advantages such as larger genome, high infectivity and capability of stable gene transferring, making them a good candidate for efficient transduction of MSCs (65) (Table 3). Nonetheless, it has been known that the induction of insertional mutagenesis in host cells caused by LVs is an important and undesired effect which limits the general use of these vectors in cancer gene therapy. However, these vectors are generally accepted for their better safety profile and lower oncogenic impact compared to other viral vectors. Though, in the next generation of lentivirus vectors, these problems have been largely resolved (66). In some techniques, modified LVs called non-integrating LVs (NILV) have been used for stable and safe gene delivery resulting in long-term expression of the transgene. For blocking the integration of the LVs into the genome of the host cell, few mutations in viral integrase coding sequence are enough to inactivate the integrase function while preserving its role in expression of the transgene (86). An engineered NILV containing green fluorescent protein coding sequences was transferred into the bone marrow-derived hematopoietic stem cells then the cells were transplanted to lethally irradiated mice. After months, the results showed the presence of the NILVs constructs in spleen colonies. The constructs were retained and detected even in the evaluations one year after the primary transduction. These results indicate the persistence of the NILVs constructs not only in the primary mother cells but also in several later generations and the progenies differentiated from the primary stem cells (87). In this respect, another advantage of LVs over other viral vectors is that they are capable of transducing non-dividing cells (66) (Table 3). This superiority becomes more significant when the fact that a notable proportion of stem cells has been considered to be quiescent (non-dividing) is taken into account (88). Moreover, by a procedure termed as the pseudotyping, wild-type lentiviral envelope glycoprotein was replaced by the adhesive domain or protein of another subtype of a virus with higher affinity to host cells receptors. The resulting viral particles can infect host cells more efficiently (89) and be stable for longer duration (90).

### Retroviral Vectors

In numerous studies, the investigators have practiced the retroviral vectors for transduction of MSCs (65) (Tables 1 and 2). Wild-type retroviruses such as moloney murine leukemia virus (MoMLV) as the name implies, have been known for their oncogenic properties due to their capability to induce insertional mutagenesis (66) (Table 3). Nevertheless, the good tropism of retroviruses to the host cells formed the basis for developing the replication-defective retroviral vectors (66). Two commonly used oncoretroviral vectors for transduction of MSCs are Mo-MLV-based and murine stem cell virus-based vectors (65). Despite an early reception of large attention to retroviral vectors, nowadays the clinical use of these vectors has been declined because many difficulties have been found to be associated with these vectors such as the absence of long-term transgene expression, ineffective transduction of MSCs, induction of insertional mutagenesis, and the requirements for administering high loads of vectors in several rounds to transduce host cells (66) (Table 3).

### Adeno-Associated Virus (AAV)-Based Vectors

Application of these vectors has been limited regarding their very low aptitude for MSCs transduction. Their weakness for transduction of MCSs prevents the extensive use of these vectors in MSCs-based cancer gene therapies (Table 3). Although an ultraviolet light-activated transduction system has been developed to increase the transduction efficiency of these vectors, but clinical applicability of AAV vectors still remained doubtful (65, 66).

### Other Viral Vectors

Although a promising transduction efficacy of up to 95% was gained using herpes virus saimiri-based vectors (HVS-V). However, there are also some difficulties in finding a laboratory friendly way for the expansion and production of replication-defective yet with high transduction efficiency HVS-V. These problems have prevented the extensive clinical use of these vectors (67) (Table 3).

### Non-Viral Vectors

Non-viral vectors such as plasmids have been utilized as another appropriate candidate for gene delivery into MSCs. The non-viral plasmids are characterized by numerous benefits in comparison to the viral vectors including the easy synthesis, low immunogenicity, high cell/tissue specificity, and no limitation in sequence size (66, 68) (Table 3). Traditional transfection methods (e.g., thermal shock) are used for transferring the non-viral vectors into the host cells (e.g., MSCs) but disappointing transfection efficiency has been achieved. The transfection methods usually lead to a high ratio of mortality (68). Nonetheless, in a novel method developed by Song et al., these problems have been mainly resolved. The method is based on employing the electric field prompted molecular vibration to transfer the plasmid DNA (pDNA) into the MSCs. The method has been characterized by some notable benefits such as the high transfection efficiency, low cell mortality and no interference with the normal activities of the cells (91). Moreover, other options of transfection-mediating methods have been advanced in which each provides some strengths besides some weaknesses. In this regard, an improved method has been introduced for constant transfection of MSCs with the help of electric power termed as the electroporation (92). In the proposed method, MSCs were transfected with pDNA using the electroporation technique, which resulted in a high ratio of successful transfection and constant expression of the transgene. Therefore, pDNAs can provide an easy transfection procedure while preserving the proper biological properties of the host cells (92). A novel method for efficient transfection of MSCs has also been recently developed based on therapeutic ultrasound (TUS). A plasmid containing an angiogenesis suppressor gene (pPEX) was transferred into the MSCs (pPEX-MSCs) by low intensity and moderate frequency TUS stimulation. The stemness, surface markers and homing properties of MSCs remained intact. The results were promising; 70% inhibition in tumor growth was achieved by just a single I.V. injection of pPEX-MSCs to mouse models of prostate cancer (93). Briefly, it could be pointed out that in recent years an exceptional progress in the field of gene delivery modalities occurred by introducing the various innovative modified non-viral vectors with the purpose of effective and perfect gene delivery (Table 3). These carriers provide several advantages such as; easy synthesis, cell/tissue specificity, low immunogenicity, and unrestricted plasmid size (65, 66). Thus far, several types of non-viral delivery systems have been trialed successfully including; calcium phosphate, microbubbles, liposomes, niosomes, nanoparticles, nano-emulsions, spermine–pullulan, magnetic-directed, and antibody/ligand-conjugated delivery systems (68, 94–97) (Table 3).

### Other Strategies of Equipping MSCs for Targeted Gene-Therapy

Given the lack of a generally accepted method for safe usage of MSCs with minimal toxicity and harmful effects (5, 6), there is still crucial requirement in developing novel targeted therapy methods with high toxicity against tumor cells while maintaining their safety in touch with normal cells. However, MSCs are the stem cells with high capacity of differentiation to various types of cells. Since the high capability of differentiation, serious concerns have been raised about the possibility of converting MSCs to tumor cells, particularly under the impression of the tumor milieu (5). Moreover, if the surface markers of allogeneic MSCs cannot match perfectly with the host immune system then an opposite reaction by immune system and subsequent elimination of transplanted/administered MSCs will be unavoidable. In addition, MSCs are relatively large cells and in the case of confronting the immune system, they can be readily detected and phagocytized by the immune cells (5). In accordance, MSCs can also be internalized by cannibal tumor cells which then direct the tumor cells to enter dormancy. The dormancy of tumor cell is now thought to be one the major reasons responsible for a phenomenon called tumor relapse (98). Furthermore, the deliverance of genetic material either by viral or non-viral vectors can increase the chance for induction of insertional mutations in MSCs or in accidentally targeted cells such as normal cells. Wrongly targeting the normal cells instead of tumor cells can end in loss of the proper functions of the cells or even conversion to the cancerous cells (5, 6).

On the opposite side, a series of solutions have been raised regarding the mentioned concerns. This implies that intact MSCs itself can act as the drugs due to their internal capability of secretion of therapeutic anticancer agents (3). Thus, administration of intact and unmodified MSCs can be just enough therapeutic against simple non-extensive injuries or non-aggressive tumors at their primary stages (3). Additionally, a surprising characteristic of MSCs is recently discovered; ability to intake the drug particles without being damaged and subsequent gradual release of the drug within the tumor after migration to the tumor site(s) (6). This property makes the MSCs needless to be genetically modified and deliver the exotic and possibly mutation-inducing vectors (6). In addition, MSCs can produce large amounts of the vesicles containing therapeutic agents with paracrine-like actions against tumor cells. Therefore, the supernatant of MSC’ culture medium or their purified extracellular vesicles can be used for cancer therapy instead of the risky injection of the MSCs into the body (99). Also, some promising novel genome editing tools have been developed recently. The methods are based on using the proteins that can recognize the specific sequence(s) on the genome. Generally, the protein is also accompanied by a nuclease to cut the recognized sequence. To date, three types of these systems including ZFNs, TALENs, and CRISPR have been developed. These systems provide accurate and sequenced-specific genome editing and can be practical in reliable delivery and integration of a therapeutic gene into the genetic material of MSCs without the risk of induction of undesired mutations (1). Alternatively, a method based on making a decoy comprised of nano-sized membranes of MSCs containing the therapeutic drugs has been advanced recently. In this method, the anticancer agents are loaded into the surface marker-consisted membranes of MSCs. Then the membranes are homogenized into the nano-sized vesicles which are termed as nano-ghosts. The MSC-derived nano-ghosts have the advantage of completely being safe due to the inability to carry any genetic material. However, these nano-ghosts also inherit the superior property from their mother cells which is the specific tumor tropism (100). Another vector-free method is based on loading the mRNAs into the stem cells instead of genetic modification with the advantage of bringing the probability of insertional mutation(s) to near zero. This occurs because unlike the DNA-based methods which are done by the integration of exotic genetic material into the genome in nucleus, the mRNAs are needless to be sent into the nucleus and are translated directly within the cytoplasm into the therapeutic proteins. However, the rapid degradation and instability of mRNA limit the broad use of this method (101). Recently, an alternative method of indirect gene therapy using small nucleotide molecules interfering with or controlling the gene regulation was also proposed (102). These gene silencing methods which are based on the small RNA (sRNAs) molecules are called RNAi technology. Three types of RNAs are involved in gene silencing including siRNA, short hairpin RNA (shRNA), and miRNA. All molecules, at the end of a series of conformational changes by the specific proteins (DICER, RISC) are converted to a complex of sRNAs-RISC which then specifically binds and cleaves the targeted complementary mRNA. The shRNA can be delivered on a plasmid to host cell (103). However, cell-based delivery systems such as MSCs can also be used to deliver these molecules to knock down an oncogenic gene as a cancer therapy. The viral vectors containing or inducing these molecules can also be employed directly for transfection of the tumor cells or within a cell vehicle (e.g., MSCs). The sRNAs, sRNA-carrying plasmids, or viral vectors containing sRNAs can be secreted as extracellular vesicles, exosomes, or virtosomes by MSCs (104, 105). Additionally, a method was developed to induce anticancer action of transgene MSCs specifically within cancerous tissues not in other tissues which MSCs may also migrate into them. In this method, a theranostic gene (a gene with dual therapeutic and diagnostic function) under the control of a RANTES-CCL5 promoter is delivered into the MSCs. The idea behind this method is originated from the fact that MSCs express the RANTES chemokine when they are within the tumor microenvironment. Additionally, the gene encoding the sodium iodide symporter (NIS) protein is employed as the theranostic gene. The MSCs carrying the NIS were injected to an animal model of metastatic colon cancer, and then a radioisotope of iodine was also injected to mouse models. The NIS protein mediates uptake of radioactive iodine into the tumor tissue which leads to subsequent sequestration of the tumor growth. This method provides a tumor site-directed anticancer therapy without any side effects on normal tissues (106). Same method was also successfully used for the treatment of xenografts of hepatocellular carcinoma (HC) in mouse. Linking the activation and expression of NIS to tumor stroma of HC resulted in tumor-specific therapeutic action and, therefore, significant inhibition of tumor growth (107). Moreover, in the case of preventing the rejection by immune system, it was suggested to use autologous MSCs rather than allogeneic or heterologous MSCs. However, another option to overcome the challenge of redundant immune responses against injected MSCs is a procedure so-called selective allo-depletion. The method is mediated through depletion of alloreactive T cells while preserving their activity against tumor cells (5). Furthermore, in order to overcome the cannibalization by tumor cells or internalization by immune cells, some methods based on the MSCs surface marker refinement or transduction with a cannibalism-suppressing gene, have been developed. The methods make the MSCs non-detectable by immune system or cannibalism-suppressor in contact with tumor/immune cells (98). Also, to avoid being faced with malignant transformation(s), researchers have suggested to use the BM-MSCs due to their good genetic stability but on the other hand the low anticancer or even tumorigenic effect of this type of MSC have limited their usage in cancer therapy. Therefore, umbilical cord MSCs (UC-MSCs) with the proved strong anticancer effect and more homogeneity have been proposed as the alternatives of BM-MSCs (108) (Figure 3).

## Conclusion

Mesenchymal stromal/stem cell-based therapies hold much hope in the treatment of cancers and tissue injuries in various organs. Encouraging results by the use of MSCs as carriers of therapeutic genes in the treatment of a variety of tumors have paved the way for extensive clinical use of this method. Engineered MSCs can overcome many of the problems caused by systemic injection of cytokines and antitumor agents such as the high cytotoxicity and low half-life. However, there are also some pitfalls in the usage of MSCs which lead to a significant delay in the clinical application of the MSCs. The main problem in cancer gene therapy is the lack of a suitable gene carrier and very low efficiency of transfection of therapeutic genes. The low transfection efficiency generally ends in low expression of delivered gene. For gene delivery, the viral and non-viral vectors are used to arm host cells (e.g., MSCs). Additionally, there are also some hurdles holding back, however temporarily, conquering in frontline of therapy of cancer; for instance, the lack of a technically suitable vector with high transduction efficiency and safety while being non-immunogen. These deprivations warrant further precise examinations on the vector-based targeted cancer therapy using MSCs as gene carriers. Thus further studies should be done to predispose the MSCs for clinical use. This can be achieved by standardization and improving the methods of MSCs isolation, culture conditions, gene transfer and the tumor tropism. Also, it is essential to determine the best source of MSCs for each disease, the number of cells, appropriate site of injection, and the best time for injection in the therapy of different types of cancers.

## Author Contributions

All authors contributed to the conception and the main idea of the work. FM and GV drafted the main text, figures, and tables. AB and AE supervised the work and provided the comments and additional scientific information. SA reviewed and revised the text. All authors read and approved the final version of the work to be published.

## Conflict of Interest Statement

The authors declare that the research was conducted in the absence of any commercial or financial relationships that could be construed as a potential conflict of interest.
